# Fluorescence in situ hybridization test for detection of endometrial carcinoma cells by non‐invasive vaginal swab

**DOI:** 10.1111/jcmm.17658

**Published:** 2023-01-10

**Authors:** Jörg Weimer, Martje Hüttmann, Asiyan Nusilati, Svenja Andreas, Jona Röseler, Nils Tribian, Christoph Rogmans, Matthias Bernhard Stope, Edgar Dahl, Alexander Mustea, Elmar Stickeler, Nina Hedemann, Inken Flörkemeier, Katharina Tiemann, Svetlana Magadeeva, Astrid Dempfle, Norbert Arnold, Nicolai Maass, Dirk Bauerschlag

**Affiliations:** ^1^ Department of Gynecology and Obstetrics Christian‐Albrechts‐University Kiel and University Medical Center Schleswig‐Holstein Campus Kiel Kiel Germany; ^2^ Department of Gynecology and Gynecological Oncology University Hospital Bonn Bonn Germany; ^3^ Institute of Pathology Medical Faculty of RWTH Aachen University Aachen Germany; ^4^ Department of Gynecology University Medical Center RWTH Aachen Aachen Germany; ^5^ Institute for Hematopathology Hamburg Germany; ^6^ Institute of Medical Informatics and Statistics Kiel University and University Medical Center Schleswig‐Holstein Campus Kiel Kiel Germany

**Keywords:** endometrial‐cancer, FISH, non‐invasive, predictive‐marker, vaginal‐swap

## Abstract

Endometrial cancer (EC) is the most common gynaecological malignancy with increasing incidence in developed countries. As gold standard, hysteroscopy confirms only 30% of suspected ECs. The detection of EC cells in the vagina by fluorescence in situ hybridization (FISH) after a smear test could reduce invasive procedures in the future. Using array‐based comparative genome hybridization (aCGH) on 65 endometrial carcinomas, most frequently imbalanced regions of the tumour genome were identified. Bacterial artificial chromosomes were used to generate FISH‐probes homologue to these human regions. The FISH test was hybridized on swabs specimens collected from the vaginal cavity. Samples from six patients without EC were selected as a negative control and on 13 patients with known EC as a positive control. To distinguish between benign and EC cases, the cut‐off value has been defined. A first validation of this EC‐FISH Test was performed with swabs from 41 patients with suspected EC. The most common genomic imbalances in EC are around the *CTNNB1*, *FBXW7* and *APC* genes. The cut‐off is defined at 32% of analysed cells without diploid signal pattern. This differs significantly between the positive and negative controls (*p* < 0.001). In a first validation cohort of 41 patients with suspected EC, the EC‐FISH Test distinguishes patients with and without EC with a sensitivity of 91% and a specificity of 83%. The negative predictive value is 96%. This is the first report of a non‐invasive EC‐FISH Test to predict EC in women with suspected EC.

## 1 INTRODUCTION

Endometrial cancer (EC) is the most common gynaecological malignancy in the developed countries, and its incidence is increasing.^1^ One reason for this is the increasing rate of obesity being the major risk factors over the past decades. In 2020, the European Cancer Information system indicated that the estimated age‐standardized incidence rate of EC was 28.9/100,000 in Europe and for Germany 24.8/100,000. EC is the ninth cause of cancer death for women in Europe, with an estimated mortality rate of 6.2/100,000.^2^ Approximately 90% of ECs are sporadic, and the remaining 10% are hereditary, that is Lynch syndrome.^3^ Until recently, the EC classification by Bokhman et al. was the standard, categorizing EC into two pathogenic types using clinical and histopathological variables. The more common oestrogen‐dependent endometrioid type I EC have a better prognosis than non‐endometrioid type II EC. The rarer form of type II EC (20%) comprises high‐grade tumours, which are clinically more aggressive and tend to metastasize. Normally, the later type is diagnosed in the advanced stages and has unfavourable prognosis.^4^ Recently, The Cancer Genome Atlas (TCGA) has provided a comprehensive genomic and transcriptomic analysis of 373 EC cases and described the molecular characteristics of these tumours in more detail, which allowed them to stratify EC into four distinct molecular subtypes,^5^, ^6^, ^7^ thus becoming the modern standard classification system. EC is currently diagnosed by invasive procedures requiring general anaesthesia. The American College of Obstetricians and Gynecologists (ACOG) committee states that hysteroscopy‐guided diagnosis of intrauterine pathologies is considered the gold standard.^8^ Postmenopausal bleeding is a frequent and early marker in EC patients. However, the postmenopausal bleeding can be caused by benign conditions as well. In addition, EC can be found in premenopausal women showing irregular vaginal bleeding. In studies looking at vaginal bleeding disorders, only around 10% of patients were diagnosed with EC but all patients underwent an invasive diagnostic procedure.^9^ It would be of great benefit for the patient and for the healthcare providers if a predictive marker could identify affected patients, hereby reducing the diagnostic burden. The goal of FISH detection is to reduce the number of diagnostic hysteroscopies in those cases of suspected endometrial carcinoma whose FISH results do not support suspicion. We, therefore, favour a test with the best possible specificity and a high negative predictive value. The false‐positive FISH test results would be identified as actually negative by the diagnostic gold standard of hysteroscopy.

Genomic imbalances that deviate from the normal diploid state could serve as biomarkers to identify tumour cells. Like most solid tumours, the EC is characterized by an accumulation of different genomic imbalances.^10^, ^11^, ^12^ These genomic aberrations occur very heterogeneously. Nevertheless, the EC shows recurring patterns. In EC, Kiechle et al. found the most common genomic imbalances with classic CGH in the genome regions 1p36, 20q13 and 16p13. Micci et al. describe 3q, 7p, 8q and 20q as regions of gain that occur in both serous‐papillary and clear cell EC.^13^ With the higher resolution aCGH, Wang et al. found five EC cell lines with 3p21, 5q23 or 17q regions that show more frequent gains and with 3p14 or 11q23 regions that show more frequent losses. A more extensive study on the detection of genomic imbalances carried out with a modern aCGH has not yet been published. With the information in which genomic regions the most common imbalances occur, fluorescence in situ hybridization (FISH) probes can be generated and used as biomarkers. FISH probes of 2.5 kb allow good visibility under the fluorescence microscope.^14^, ^15^ When designing larger FISH probes, microscopic visibility improves, but smaller deletions in the stained areas remain hidden. However, translocations larger than 2 kb become visible as additional signals. Thus, when choosing the FISH probe size, visibility must be weighed against the detectability of small deletions. We show that by detecting cytogenetic abnormalities in cells extracted from vaginal brushing an easily feasible detection of EC cells is possible.

## 2 MATERIAL AND METHODS

### 2.1 Biomaterial

We obtained tumour DNA from 65 endometrial carcinomas for the aCGH. Among them, 42 were type I and 23 were type II EC. All bio samples are from the University Kiel bio‐bank network p2n, the University of Greifswald bio‐bank, and Department of Hemato‐pathology, Hamburg. The tissue was provided as 30 fresh, and 35 formalin‐fixed paraffin‐embedded (FFPE) materials. As a negative control, we used one inconspicuous DNA sample from the peripheral blood for the aCGH. For the EC‐FISH Test on vaginal brush cells, we collected swab specimens from patients with suspected EC and, if possible, swabs directly from the tumour tissue. The functionality of the EC‐FISH Test was examined on smears from 22 patients, six of which had no tumour background and were used as negative controls in the FISH test. Vaginal smears from 13 patients definitely had EC. Thus, they were used as positive controls. Of these, six corresponding swabs were also available directly from the tumour. Three tumour swabs had no corresponding vaginal swabs. To validate the FISH test, vaginal swabs from 41 patients who consulted our clinic were examined. For the use of patient material, we have received an ethics vote from the ethics committee of the Medical Faculty of the Christian‐Albrechts‐Universität, Kiel (D 440/17) as well as a declaration of consent from each patient. The present study has been refined in the spirit of the current Helsinki Declaration.

### 2.2 DNA preparation

QIAamp® DNA mini kit was used to extract genomic DNA (gDNA) from fresh tissue by the manufacturer's protocol. Genomic DNA from FFPE tissue was isolated by BIOstic® FFPE tissue DNA isolation kit or QIAGEN® GeneRead DNA FFPE kit following the manufacturer's protocol after microdissection of tumour tissue out of 10 μm tissue cuts. The quantity of extracted double stranded DNA was determined by Qubit® 2.0 fluorimeter. The purity of extracted DNA was also measured by using the spectrophotometer NanoDrop™ 2000.

### 2.3 Array design

With the support of Oxford Gene Technology (OGT), we upgraded their standard CGH + SNP 4 × 180k array (1500‐KIE; design_0002 Additional*POLE; 084718*) with increased resolution in 13 genes, conspicuous in the molecular sub classification of endometrial cancer. Those genes were *ARID1A, CTNNB1, PIK3CA, FBXW7, APC, PTEN, FGR2, KRAS, POLE, BRCA2, ZFHX3, TP53 and SPOP*. In the high‐resolution gene regions, the sequences in the array samples partially overlap. Since the algorithm already evaluated four adjacent array probes beyond the threshold as an abnormality, very small abnormalities positioned next to each other could result in high‐resolution genes. Known abnormalities of the Agilent female reference DNA were removed from the evaluation as artefacts.

### 2.4 aCGH

Following the CytoSure™ Array Handbook for 4 × 180 k formats (OGT, Ver. 2), equivalent amounts (500–1000 ng) of DNA were labelled with either Cy3‐dCTP for tumour DNA and Cy5‐dCTP for Agilent female Reference DNA using the CytoSure Genomic DNA labelling kit. Before labelling, to match size of the FFPE samples, reference DNA was prepared by shearing with focused‐ultrasonicator Covaris S220. The target peak for base pair size was 150 bp–200 bp. After labelling, the concentration of each sample was measured using the NanoDrop™ 2000. DNA concentrations greater than 225 ng/μl and dye concentrations greater than 2.6 pmol/μl were considered adequate for hybridization. The labelled DNA sample were combined and then added Cot‐1 DNA (Life Technologies, cat. no. 15279–011), Agilent Blocking Agent from Oligo aCGH Hybridization Kit (OGT, cat. no. 500014) and Hybridization buffer following CytoSureTM Array Handbook (4 × 180k). The Agilent hybridization gasket was placed into an Agilent chamber base; 100 μl of the hybridization mix was pipetted onto each chamber of the gasket slide. The CGH + SNP array was placed onto the gasket slide. The clamp was positioned on the slide and tightened firmly the thumbscrew. The hybridization chamber was placed in the hybridization oven at 65°C for 22 h, rotating at a speed of 20 rpm. After hybridization, arrays were washed in Agilent washing buffers following the manufacturer's protocols. Then, the slide was dried under ozone‐free air, placed into the Agilent slide holder and scanned by the SureScan Dx Microarray Scanner (Agilent, G5761A). The scanned image was extracted using Agilent Feature Extraction Software 3.0.5.1. Data analysis has been performed using the CytoSure Interpret Software Ver. 4.8 (OGT, Oxfordshire). Annotations were based on the Human Genome NCBI database CRCh37, and the results were displayed. Gene contents of aberrations were cross‐checked with the UCSC (http://www.genome.ucsc.edu) and Ensemble databases (http://www.ensembl.org).

### 2.5 Generation of FISH probes

Conspicuous aberrant regions with the most frequent changes in the examined ECs were defined as target regions. Within these target regions, we have identified potential bacterial artificial chromosomes (BACs) that could be considered as possible FISH samples. We obtained corresponding BAC clones from BACPAC Genomics Inc (Richmond, Ca) in lysogeny broth (LB) agar stab cultures. These DH10B Escherichia coli clones were grown overnight in LB with 12.5 μg/ml chloramphenicol at 37°C and periodically shaken. BAC DNA was isolated with the Qiagen® Plasmid Mini Kit (Hilden) following the manufacturer's protocol. As described by Weimer et al., the BAC DNA was amplified and labelled with fluorescent dyes by DOP‐PCR.^16^ RP11‐349C9, RP11‐474P6, RP11‐186B18 and RP11‐178D22 were labelled with dUTP‐Cy3. RP11‐962I23, RP11‐124 K18, RP11‐60P20 and RP11‐472P14 were labelled with green‐dUTP. RP11‐90 L5, RP11‐54D8 and RP11‐61H16 were labelled with green‐dUTP and Cy3‐dUTP. The labelled PCR products were combined and supplemented with 40 μg Cot‐1 DNA (GeneOn) and precipitated in 70% ethanol overnight. The precipitate was centrifuged for 30 min at 13,000 RPI, the supernatant was discarded and the pellet was freeze‐dried. The dry pellet was dissolved in 50 μl hybridization solution (2× SSC, 50% formamide, 10% Dextran sulphate, 1% Tween 20, pH 7.0).

### 2.6 Preparation of brush cells

Simple swab brushes were used for the smears. After the swab, the breakable brush head is transferred to 4 ml Certipur buffer solution (Merck), which was poured into a 15 ml Falcon tube. This could be stored at 4°C for about 2 days until the cells were prepared. The brush head was carefully rinsed in the buffer solution and then removed. The resuspended cells were fixed by adding 1 ml of cold fixative (One part glacial acetic acid and three parts methanol) four times. The suspension was inverted in between. After the suspension has been centrifuged for 5 min at 450 g and 4°C., the supernatant was carefully removed and the pellet was resuspended with 4 ml of cold fixative. This process was repeated twice. The suspension then remained on ice for 30 min. After another centrifugation and subsequent resuspension with fresh fixative, the suspensions could be stored at −20°C. For the preparation of the smear cells, clean slides are positioned upright in a humid chamber at an angle of about 45°. Twenty μl of the suspension should run down the slide in two rows. After about 10 min of incubation in the humid chamber at 37°C, the slides dried at room temperature. Then, the slide was neutralized in a 50 ml Falcon Tube with 35 ml Certipure buffer solution for 4 min.

### 2.7 EC‐FISH Test

The slide with the smear cells was incubated for 2 min in 2 × saline sodium citrate (SSC) buffer at 37°C. The slide was then transferred to a 37°C. 0.01 M hydrochloric acid with 0.005% pepsin for 15 min. The slide was washed in phosphate‐buffered saline (PBS) buffer, pH 7.4 at room temperature for 3 min. Then, the cells were fixed in PBS with 1% buffered formaldehyde and 20 mM magnesium chloride, pH 7. The slides were washed again for 3 min in PBS at room temperature. The cells were dried in an ascending series of ethanol (70%, 85% and 100%) for 1 min each. Finally, the slides were air dried. At the next step, DNA of the swab cells and the FISH probe DNA were denatured simultaneously. Ten microliters of the FISH probe was denatured under a rubber sealed 22 × 22mm cover glass for 10 min at 75°C. Then, the slide was hybridized at 37°C. in a humid chamber upside down overnight. After hybridization, the cover slip was carefully removed and the slide was washed at room temperature for 2 min in 2× SSC with 0.1% Igepal. The next washing step was carried out for 2 min in 0.4 × SSC with 0.3% Igepal at 72°C. This is followed by another washing step in 2× SSC with 0.1% Igepal at room temperature for 1 min. The slides were incubated again in an ascending series of ethanol (70%, 90% and 100%) each for 1 min. Finally, the slides were air‐dried and covered with the mounting medium containing DAPI. The hybridization was evaluated and viewed on a Zeiss Axioplan 2 fluorescence microscope (Zeiss, Oberkochen, Germany), which is equipped with optical filter sets for FITC and Cy3. We used ISIS (Metasystems, Altlusheim, Germany) as a recording and documentation software. At least three times, 50 interphase nuclei per patient were counted and the average proportion of conspicuous signal patterns was documented. Three and a half times the standard deviation was added to the mean proportion of the negative controls, resulting in a limit value above which the presence of endometrial carcinoma cells could be detected. All signal constellations within an interphase nucleus that deviated from the normal diploid pattern with two signals per each colour (loci) were rated as conspicuous.

### 2.8 Statistical analysis

Median proportions of cells with a conspicuous signal pattern between patients with and without EC were compared using Mann–Whitney *U*‐test. To evaluate the accuracy of the FISH test for diagnosis of EC at different cut‐off values of the proportion of conspicuous cells, we calculated the receiver operating characteristic (ROC) curve, plotting true positive fraction (sensitivity) against false‐positive fraction (1‐specificity) for all possible cut‐off values. The area under the curve (AUC) of the ROC gives a measure of the overall suitability of the FISH test. We used Origin 2021 Ver. 9.8.0.200 (OriginLab Corporation), and R Statistical Software (v.4.1.0)^17^ with the pROC^18^ and plotROC^19^ packages.

## 3 RESULTS

**FIGURE 1:**
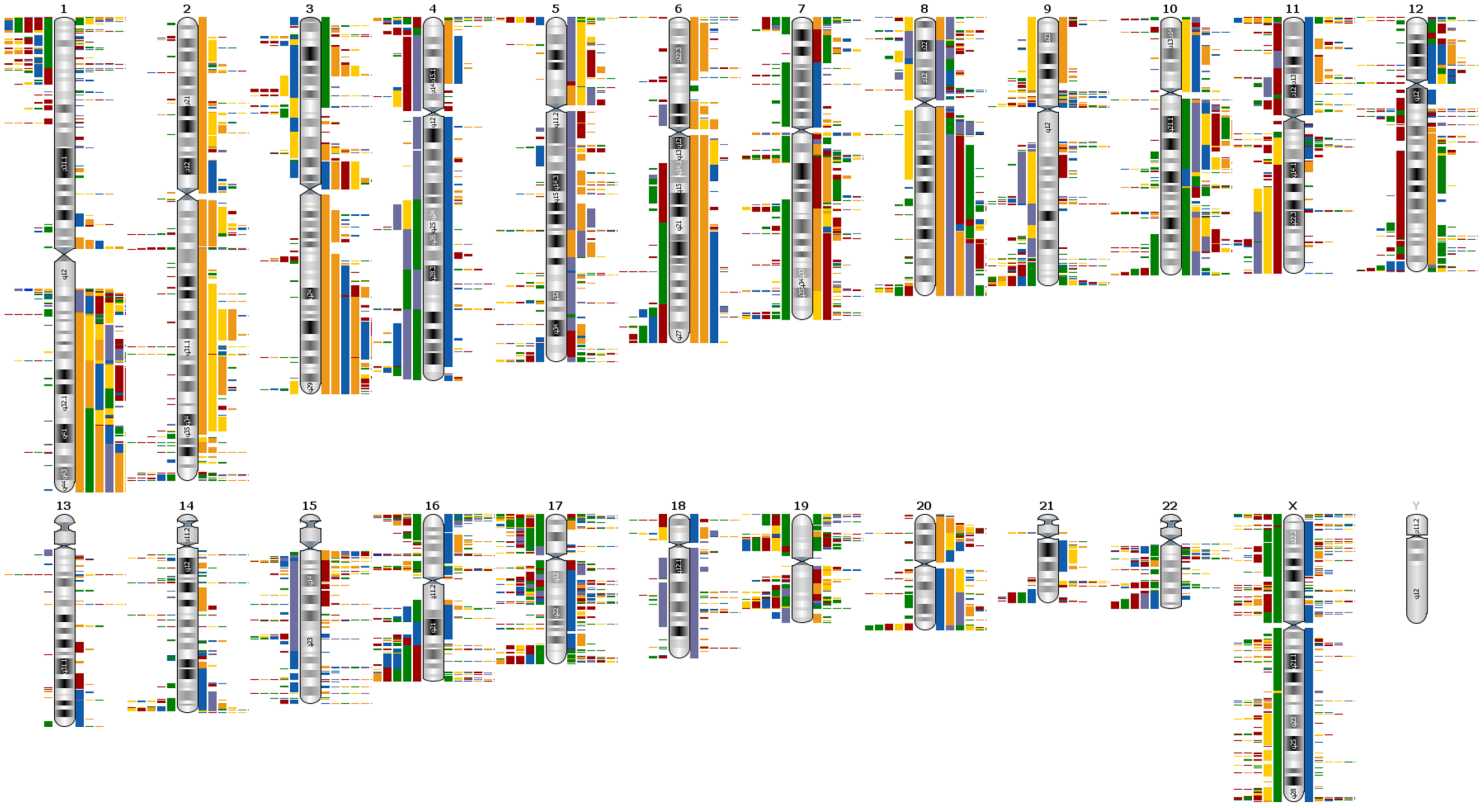
Overview of gains and losses in 65 endometrial cancers. The bars to the left of the ideograms show losses, those to the right show gains. The different colours in the bars represent the individual cases. Unfortunately, such an overview does not allow the adequate resolution that is necessary for the analysis in detail.

**FIGURE 2:**
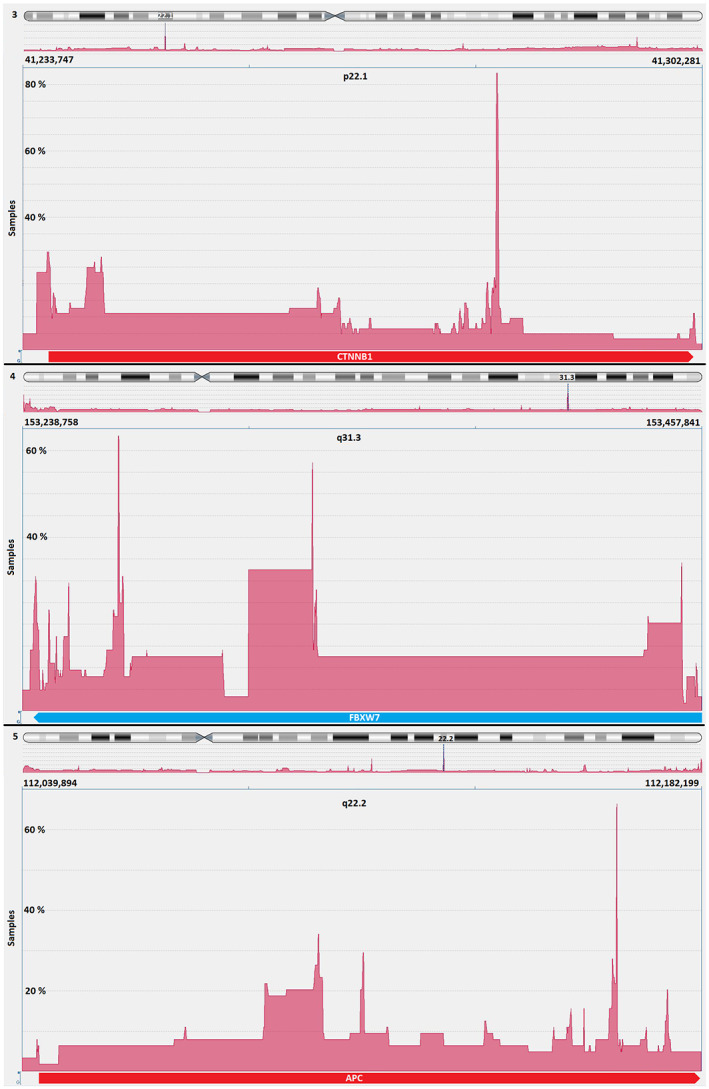
*CTNNB1*, *FBXW7* and *APC* genes are located within the three genomic regions showing the most frequent gains and losses in 65 endometrial cancers analysed in this study. Above: Most imbalances are in 83% of all cases in 3p22.1 in the *CTNNB1* gene. Middle: The smallest overlapping region with the third most common imbalances is in 4q31.3 in the *FBXW7* gene in 63%. Below: The second most common imbalance is found in 65% of all investigated cases in 5q22.2 in the *APC* gene.

In the first step, genomic regions with frequent gains and losses in all EC were analysed (Figure 1) by OGT's analysis software. It provided a comprehensive overview of regions of interest for further detailed analysis of regions with high‐frequency aberrations. The abnormalities indicated by the software were superimposed, allowing to identify regions of interest with the most gains and losses. In principle, gains and losses within the same tumour can be detected side by side in these regions. This software driven population analysis function allowed high‐resolution detection of the smallest overlapping imbalances. The most noticeable region is in chromosome 3p22.1 at the nucleotide location from 41,280,499 to 41,282,752. Interestingly, this region includes the final exons of the *CTNNB1* gene. 83% (54/65) of all analysed ECs presented general imbalances in this 2.25 kb region. Among them, there were 65 gains and only 3 losses. The location with the second most common abnormalities in our EC cohort was detected on chromosome 5q22.3 between the nucleotides 112,162,948 and 112,164,590. In this region, with an extension of 1.6 kb around Exon 11 of the *APC* gene, 42 imbalances (65%) were detected in this cohort with 48 gains and 1 loss. A further common area was detected on chromosome 4q32.1 between the nucleotide location 153,242,171 and 153,271,571 (29.4 kb) and covering *FBXW7* gene in 63% (41/65) of examined endometrial carcinoma. There were 111 gains and 10 losses within this region in the 65 cases (Figure 2). In known databases published to date, all three regions show no or very few copy number variations (CNVs). The range of the most common abnormalities extended just over 300 base pairs. A cross‐hybridization due to matching DNA sequences could be ruled out by comparing sequences with the BLAT tool (Human (hg19) BLAT Results (ucsc.edu), https://genome.ucsc.edu/cgi‐bin/hgBlat).

**FIGURE 3:**
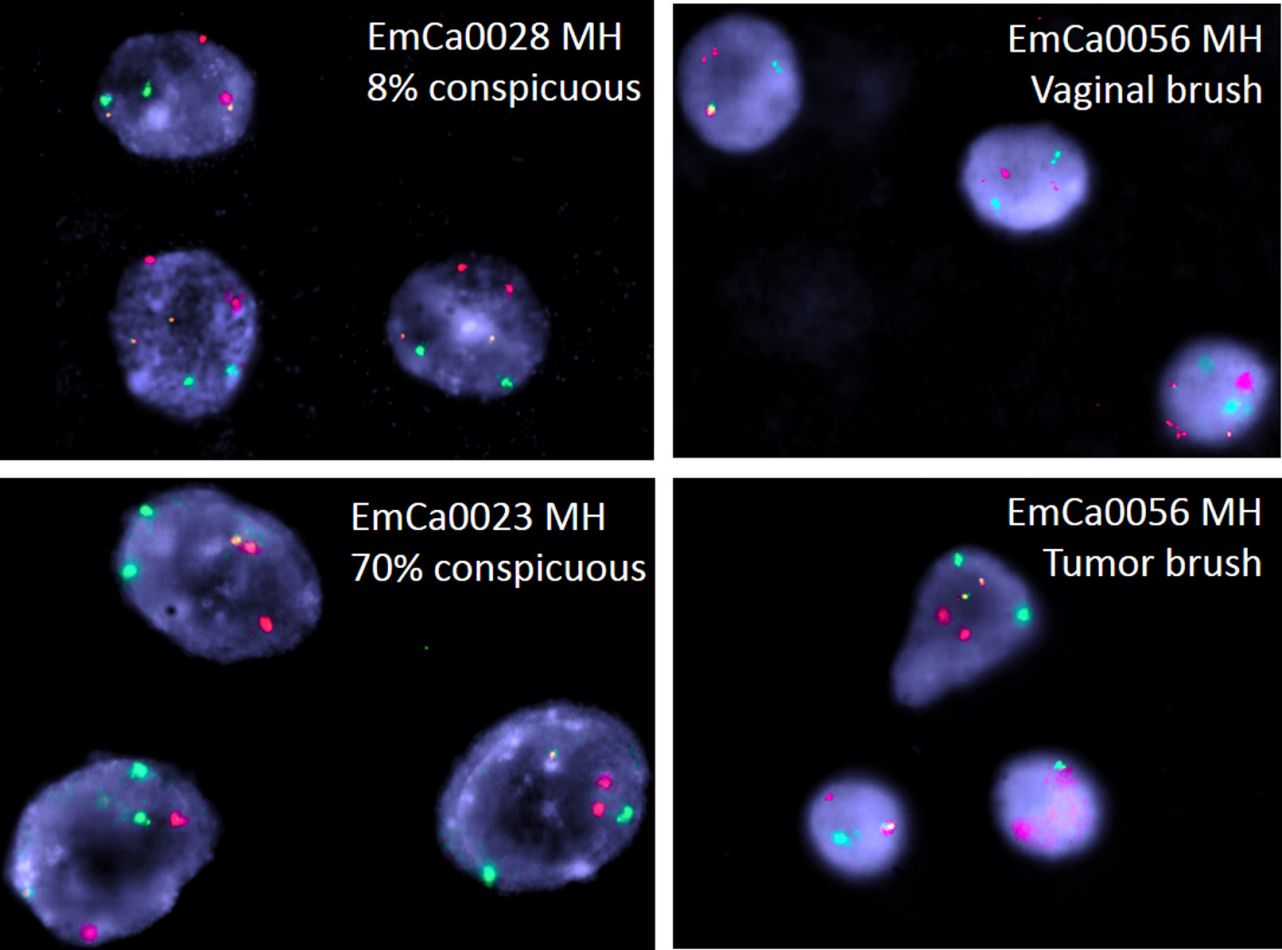
Exemplary interphases from the validation cohort. red = 4q31.3; green = 5q22.2; yellow = 3p22. Above, left: EC‐FISH Test negative case. Bottom, left: EC‐FISH Test positive case. Right: EC‐FISH Test positive case with a vaginal swab (above) and a swab from the corresponding tumour (below).

**FIGURE 4:**
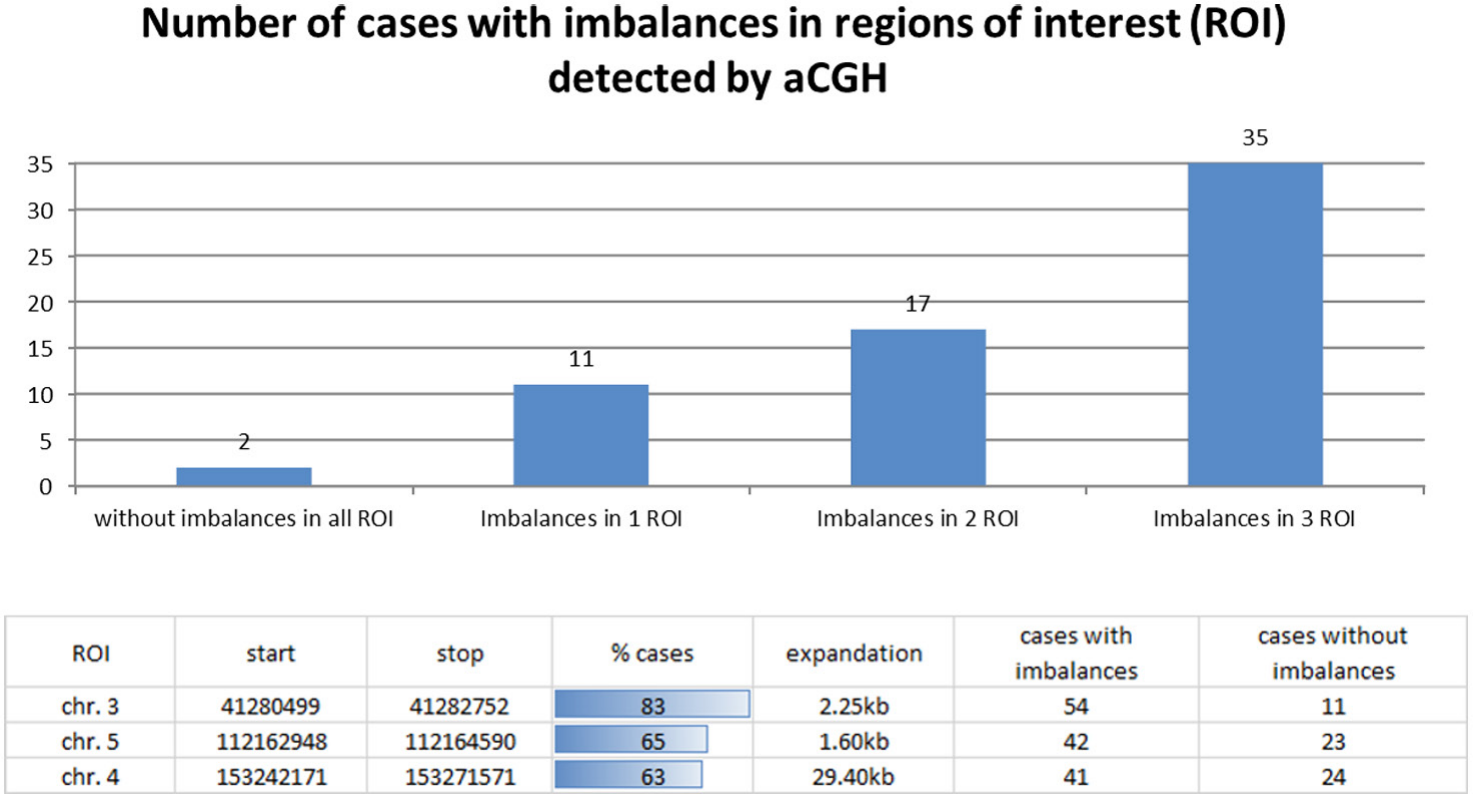
Distribution of the measured imbalances in the three regions with the most common imbalances. Above: The graphic shows the frequency of imbalances in different combinations within the three regions of interest (ROI) based on the aCGH data. Below: The table shows the relative and absolute number of imbalances and their extent in the three most conspicuous regions of interest.

Interestingly, there was no difference in the pattern of aberrations for these three areas when comparing endometrial cancer type 1 and type 2. If the detection of abnormalities in these three regions was based on the genomic imbalance findings using the aCGH technique, and if only one abnormality per region was evaluated as evidence of endometrial carcinoma cells, we could calculate the test's theoretical sensitivity. Under the microscope, FISH signals with a minimum size of 2 kb can be detected reliably. In principle, imbalances can be detected by a FISH test, considering they are not too small. Examples of EC‐FISH Probe hybridizations at interphases for validation are shown in Figure 3. The significance of aCGH in detecting EC is a degree of potential effectiveness of the EC‐FISH Test in principle. An abnormality in one of the three regions should indicate a tumour cell. Thirty‐five aCGH analyses of ECs show imbalances in all three regions: 17 cases in only two regions and 11 cases in only one region. For two cases, no abnormalities were detected by aCGH in any of the three regions (Figure 4), resulting in a hypothetical sensitivity of 97%.

**FIGURE 5:**
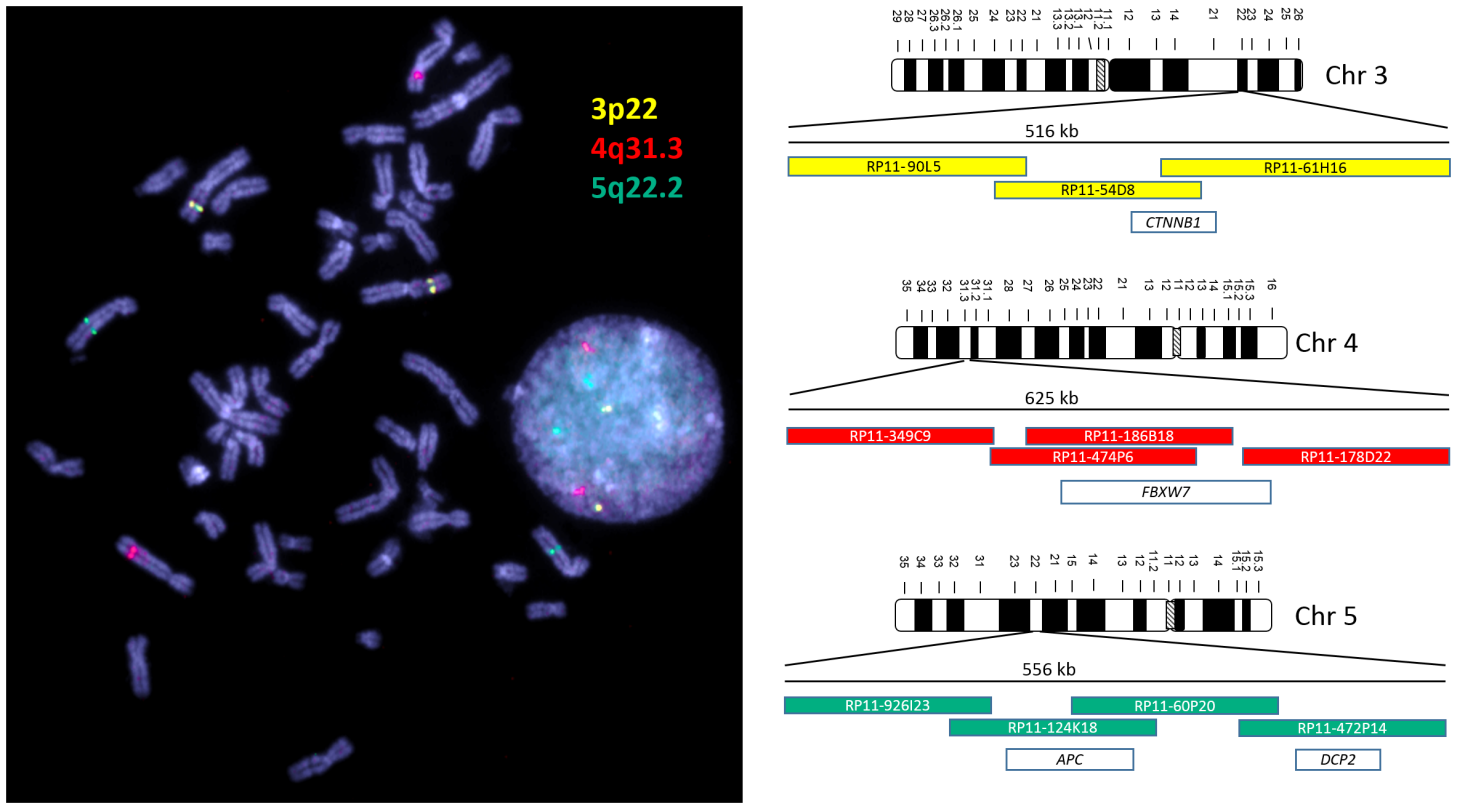
Metaphase and interphase nuclei from lymphocytes are shown on the left side. On the right are the three ideograms of the chromosomes on which the EC‐FISH test loci are located. The coloured bars outline the BAC clones used. These are coloured according to the fluorescence colour and positioned relative to each other. The number above the BAC clones indicates the extent of a regular FISH signal. The white bars represent genes covered by the FISH signal. The dye signals correspond to the differently coloured areas of the EC‐FISH Test probe with which endometrial cancer cells are identified if the pattern deviates from the normal distribution shown here.

**FIGURE 6:**
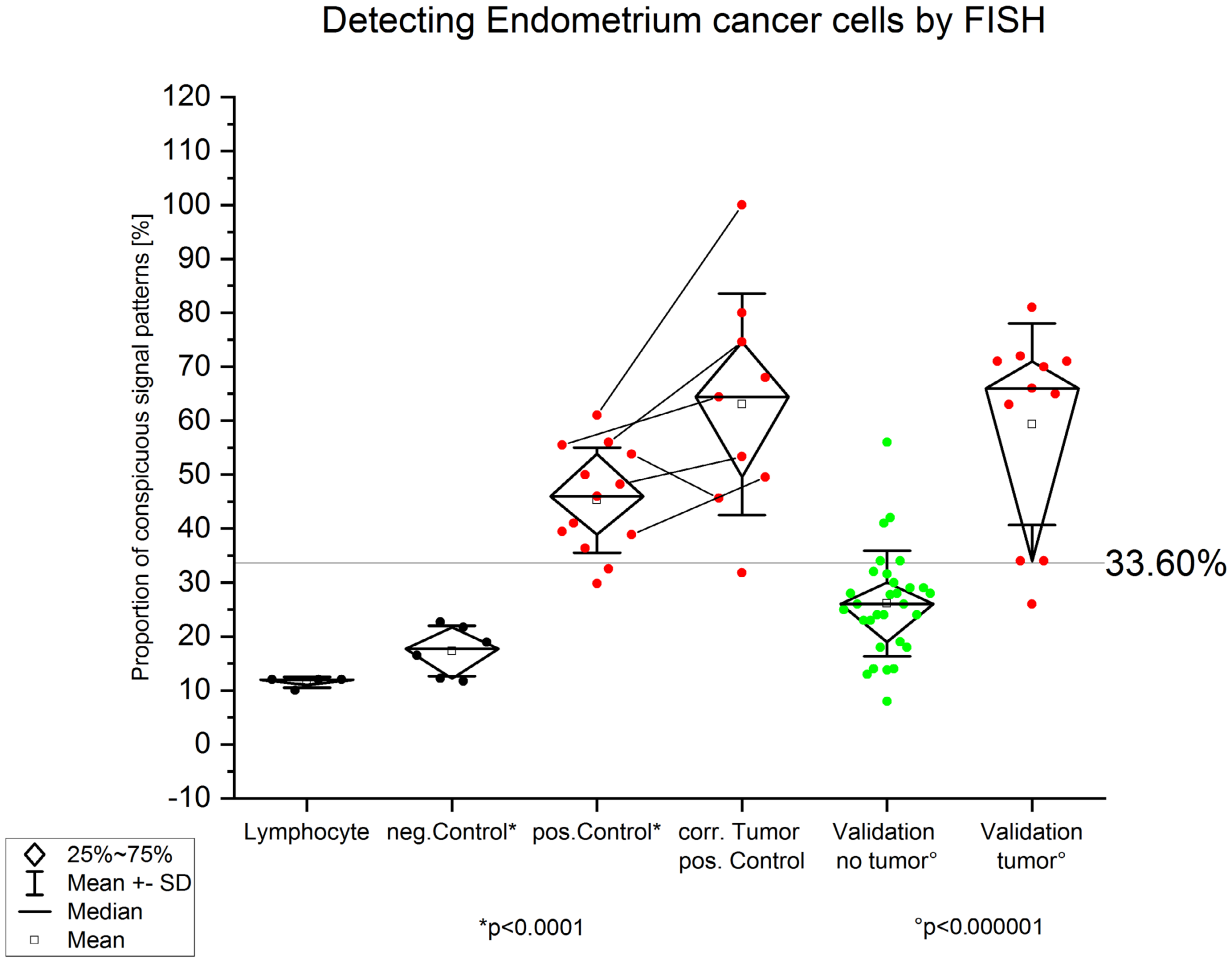
Graphic shows the percentage of FISH signal patterns after hybridization with the generated EC‐FISH Test. Left: Hybridization on male, normal lymphocytes to demonstrate the function of the EC‐FISH Test. Next to it on the right: Hybridization of the EC‐FISH Test on vaginal smears that have no tumour abnormalities. Centre left: Hybridization of the EC‐FISH Test on vaginal swabs taken immediately before surgery from patients with endometrial cancer. With *p* < 0.0001, a two‐sample *t*‐test shows a significantly different value distribution between negative and positive controls. The mean value of the negative control plus three and a half times the standard deviation defines the limit value at 33.60%. Right, in the middle: hybridization of the EC‐FISH Test to smears taken directly from endometrial cancer. The lines between the dots indicate corresponding vaginal and tumour smears. Right: Validation on 41 vaginal swabs from patients with suspected endometrial cancer. The EC‐FISH Test results on smears from patients whose suspicions were not confirmed after hysteroscopy are shown here in green. Those whose suspicions have been confirmed are shown in red on the far right. The distribution between both groups differs significantly according to the *t*‐test with *p* < 0.000001.

**FIGURE 7:**
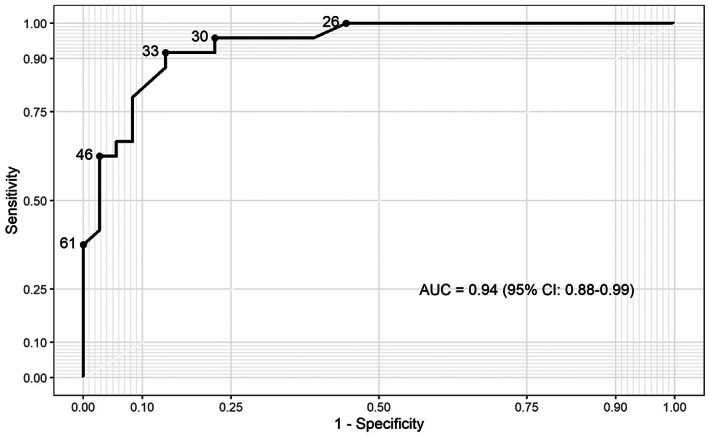
ROC curve of EC‐FISH Test determined from all examined ECs in this study.

Thus, we used overlapping BAC clones around the *APC* (5q22.2), *FBXW7* (4q31.3), and *CTNNB1* (3p22.1) genes to generate FISH probes (a total size of 516–625 kb) covering the most conspicuous regions. Different coloured FISH probes were generated using these BAC clones to mark those regions of interest separately. The three differently coloured loci of the EC‐FISH Test were hybridized together. To determine the signal intensity and the expected position, the probes were tested on interphases of normal male lymphocytes (Figure 5) showing two signals per probe for one chromosome type. To verify whether abnormal cells can be detected in the smear using the EC‐FISH Test, the cut‐off for the test had to be determined first. The evaluation of six hybridizations on vaginal swabs from patients without tumours revealed a deviation of the expected signal pattern from two signals per colour in a mean proportion of 17.29% (SD 4.66%, median 17.95%) of the tested cells. To increase the test specificity, we added 3.5 times the standard deviation value to the mean, resulting in a cut‐off of 33.60% (Table 1). In cases where the pathologic signal pattern occurred with a frequency >33.60% of cells in the vaginal smear, detection of EC was very likely. In comparison with the negative controls, 30 vaginal swabs from patients with confirmed endometrial cancer (positive controls) showed a mean proportion of 45.28% (SD 9.76%, median 46%) of cells with a suspected signal pattern. Thus, the proportion of suspicious cells is significantly higher in vaginal swabs from patients with EC than in control swabs from patients without EC (*p* < 0.0001, Mann–Whitney *U*‐test). To show that the FISH probe actually indicate endometrial carcinoma cells, we hybridized the FISH probe directly to smears of tumour material and evaluated each three times with 50 cells. Next, we investigated the proportion of cells with a conspicuous signal pattern in vaginal brushes and corresponding smear retrieved directly from the tumour, showing higher numbers in five brushes of corresponding tumours and lower in one. The mean proportion of cells with a conspicuous signal pattern in direct tumour brushes was 63% (Table 1; Figure 6). In our validation sample, 11 out of 41 patients were histologically proven to have carcinoma uteri (Table 2). The remaining 30 had no tumour background. Of those with a tumour background, one sample was not identified as EC by the EC‐FISH Test (median proportion of conspicuous cells 66%; mean 59.36% [SD 18.71%]). Of the 30 patients without a tumour background, five had a proportion of conspicuous signal patterns that was above the 33.60% and thus falsely indicate EC (Table 2; Figure 5). The signal distribution of the 10 true positive cases (Table 2) is available online in an additional file from a third of all evaluated interphases. Using a cut‐off value of 33.60% results in an estimated sensitivity of 0.91 and a specificity of 0.83 using all initial and validation sample together. The negative predictive value of this test is 0.96. The ROC curve (Figure 7) shows sensitivity and specificity for different possible cut‐off values and has an AUC of 0.94 (95% CI 0.88–0.99), indicating a very high accuracy of FISH test for diagnosis of EC.

**TABLE 1:** Calculation of the cut‐off. The proportions of conspicuous signal patterns in the vaginal smears of the negative controls as well as in those of the positive controls and the tumour smears are compared here. The mean value and the standard deviation are calculated below. The cut‐off of this EC‐FISH Test is calculated from the sum of the mean value and three and a half times the standard deviation.

	Conspicuous signal pattern (%)		
	Vaginal brush		Tumour brush
Cases	Neg.control	Pos. control	Tu‐control
EmCa0036	21.71		
EmCa0037	22.69		
EmCa0038	11.69		
EmCa0039	12.22		
EmCa0040	18.95		
EmCa0041	16.47		
EmCa0002		56.00	74.60
EmCa0007			80.00
EmCa0009		29.81	
EmCa0011			68.00
EmCa0012		32.56	
EmCa0014			31.81
EmCa0015		46.00	
EmCa0016		50.00	
EmCa0017		36.36	
EmCa0020		38.89	49.54
EmCa0024		41.03	
EmCa0022		48.21	53.33
EmCa0029		39.47	
EmCa0031		53.81	45.67
EmCa0047		55.47	64.41
EmCa0049		61.02	100.00
Standard deviation (SD)	4.66	9.76	20.52
Mean proportion	17.29	45.28	63.04
Cut‐off: mean + (3.5*SD)	33.60		

**TABLE 2:** EC‐FISH Test validation result. Comparison of the detected percentages of conspicuous EC‐FISH Test patterns to the patient's clinical situation.

Cases	Conspicuous signal pattern (%)				
	False Positive	True Positive	False Negative	True Negative	Clinic
EmCa0003 MH				27.78	No tumour
EmCa0004 MH				13.79	No tumour
EmCa0005 MH				24	No tumour
EmCa0008 MH		34			Cervix carcenoma
EmCa0010 MH				28	No tumour
EmCa0013 MH				31.58	No tumour
EmCa0018 MH				18	No tumour
EmCa0019 MH				24	No tumour
EmCa0021 MH	34				No tumour
EmCa0023 MH		70			Uterus Sarcom
EmCa0025 MH				30	No tumour
EmCa0026 MH			26		Endometrial carcinoma
EmCa0027 MH		66			Uterus carcinoma
EmCa0028 MH				8	No tumour
EmCa0030 MH		72			Cervix carcenoma
EmCa0032 MH				14	No tumour
EmCa0033 MH				14	No tumour
EmCa0034 MH				32	No tumour
EmCa0042 MH				26	No tumour
EmCa0043 MH				23	No tumour
EmCa0044 MH				19	No tumour
EmCa0050 MH				29	No tumour
EmCa0051 MH				23	No tumour
EmCa0052 MH				13	No tumour
EmCa0053 MH				24	No tumour
EmCa0054 MH		34			Uterus carcinoma
EmCa0055 MH		71			Uterus carcinoma
EmCa0056 MH		63			Uterus carcinoma
EmCa0057 MH				26	No tumour
EmCa0058 MH				29	No tumour
EmCa0059 MH				28	No tumour
EmCa0060 MH		81			Uterus and cervix carcinoma
EmCa0061 MH	34				No tumour
EmCa0062 MH	42				No tumour
EmCa0063 MH		65			Endometrial carcinoma
EmCa0064 MH		71			Uterus carcinoma
EmCa0065 MH				28	No tumour
EmCa0066 MH				18	No tumour
EmCa0067 MH	41				No tumour
EmCa0068 MH				25	No tumour
EmCa0070 MH	56				No tumour

## 4 DISCUSSION

To our knowledge, this is the first study on using aCGH as a tool to detect EC in women with suspected EC by transvaginal ultrasound. Using aCGH the chromosome region, 1q23 was identified as the most frequently gain in EC.^20^, ^21^ In our opinion, Morrison et al. carried out an extensive work on EC, although the differentiation was made exclusively on the tumour grading.^21^ Based on their work, a FISH probe designed by Morrison and colleagues showed a significant correlation of this overrepresentation with the tumour grading and ultimately with overall survival in an extensive collective. Patients with a dedifferentiated EC had a significantly reduced overall survival if they had multiple copies of chromosome region 1q23. This shows encouraging evidence how aCGH data allow the development and application of clinically relevant FISH probes. From the wealth of data made available by the aCGH, only those loci in the human genome should be initially considered as potential markers that deviate significantly from the normal diploid state of healthy cells. In order to draw conclusions in this regard, especially in gene regions that are relevant to EC, an array was designed with a particularly high resolution for these regions.^6^ With a low‐resolution aCGH, Fles et al. were able to partially confirm Kiechle's et al. EC imbalances on paraffin‐fixed DNA material without pointing out specific, gene‐related hotspots.^10^, ^22^ However, their work confirms the imbalances we found most frequently occurring in genomic regions of EC, except chromosome five. Wang et al. 2010 also found imbalances by aCGH in regions that we have declared as gain^11^ in our previous EC data. In the aCGH analysed ECs, the *CTNNB1* gene on chromosome 3p22.1 was found to contain 83% of imbalances. The smallest overlapping region was limited to only 300 bp (see Figure 2). Such a small expansion, of course, cannot be detected by FISH. Nevertheless, the more extensive imbalances, which in individual cases contribute to this smallest overlapping region and extend over several kb, can very well be detected with the EC‐FISH Test. The protein encoded by *CTNNB1* is involved in a protein complex that forms the adherent junctions (AJs). The importance of *CTNNB1* for the development of EC has been widely studied.^23^, ^24^, ^25^, ^26^ Terakawa et al., 2019 show that mutations in *CTNNB1* can drive myometrial invasion in an endometrial carcinoma mouse model.^27^ With over 60% of imbalances in the *FBXW7* gene in chromosome 4q31.3, this region is the second most common in our study. FBXW7 is involved in the controlled breakdown of proteins in cells. Liu et al. were able to show that suppressed expression of *FBXW7* in EC leads to the proliferation of tumour cells.^28^ Cuevas et al. 2020 suspect *FBXW7* to be involved in mesenchymal‐epidermal transition in endometrial carcinoma.^29^ The third most common imbalance in our study was found also in more than 60% of the ECs examined by aCGH in the *APC* gene in chromosomes 5q22.2. The *APC* gene is a well‐known tumour suppressor gene, associated with *CTNNB1*, with diverse functions, that is cell migration and adhesion. Ignatov and co‐authors associate hyper methylation of the *APC* gene as an early event with the development of EC.^30^ Moreno‐Bueno and colleagues could not show a functional equivalent to mutations in *APC* in EC.^31^ The imbalances we detected in these three loci of EC with tumour‐relevant genes reinforce the assumption that a gene dose effect could influence tumour development. A directly proven impact of the genes on the molecular genetic classification^6^ of EC cannot be derived, but their claim is also different. The loci described in their work represent the most common imbalances in EC in our study. Therefore, these loci should serve as biomarkers for the detection of EC cells. The justification for a simple EC detection test can be found in the currently invasive diagnostic procedure to confirm EC. In nearly half of the suspected EC cases, the diagnosis is not confirmed.^32^ About a third of the patients show polyps instead of EC and 13% have myomas. Developments that allow non‐invasive detection of EC, such as using ultrasound or pipelle, have not asserted themselves as the gold standard.^33^, ^34^, ^35^, ^36^ A simple test using a smear, as is already the case with cervical cancer, could alleviate the currently standard burden of diagnostic hysteroscopies in patients with the suspicious ECs. The detection of gains or losses in at least one of the focused three chromosomal regions in EC by aCGH seems very sensitive (97%) to the tumour material. As shown in Figure 4, only 2 of 65 (3%) examined cases have no deviation from the typical two signals in all three loci.

The purpose of the informative value of various FISH tests is adapted to the different questions and the genome to be tested. FISH tests for the diagnostic detection of specific chromosomal rearrangements, such as gene fusions or gene breaks, are often detected in lymphomas or leukaemias by break‐apart or fusion probes.^37^ In these cases, exact genetic conditions are sought that can have a therapeutic effect. The cut‐off for a reliable statement is often very low for such FISH tests. This is favoured by the significantly lower accumulation of chromosomal aberrations in lymphoma and leukaemia than in the solid tumour cases.^38^, ^39^, ^40^ Toujan et al. 2009 examined 28 cell lines of Burkitt's lymphoma and found a median of four copy number alterations (CNAs) per lymphoma cell line.^41^ Significantly more chromosomal aberrations accumulate in gynaecologic solid tumours, particularly when the DNA repair systems that so often fail are impaired.^11^ While Burkitt's lymphomas have CNA median of only about 4, endometrial cancer has a CNA median of 15.^11^, ^41^ With a higher number of rearrangements in the tumour cells, a more frequent involvement of the marked FISH loci can also be assumed. For example, astrocytoma, as solid tumours with a large number of CNAs,^42^, ^43^ also have a cut‐off of over 30% for a prognostic FISH test that detecting homologous deletions.^44^ The high cut‐off of the EC‐FISH Test is also due to the high proportion of CNAs in the endometrial carcinomas, as Figure 1 confirms. In connection with the chromosomal heterogeneity of the EC, a ploidy standard close to any centromere as an additional FISH signal cannot bring any gain in knowledge.

The validation of the EC‐FISH Test does not clearly confirm the suspicion of endometrial carcinoma. In five suspected cases, the EC‐FISH Test supports this suspicion and can only be ruled out by a hysteroscopy. In contrast to these false‐positive cases, the EC‐FISH Test does not confirm one suspected case of endometrial cancer, even though the patient has endometrial cancer. As already mentioned, not all suspected cases of endometrial cancer are confirmed by hysteroscopy. Many patients consent to this unpleasant and time‐consuming procedure in vain. The importance of the EC‐FISH Test lies in the possibility of supporting the suspicion, to spare some women the hysteroscopy, if the suspicion is not supported. A hysteroscopy would rule out the suspected tumour in false‐positive cases. Although these patients are subject to the additional burden of a hysteroscopy, the clinical consequences do not go beyond the dangers of this procedure. The false‐negative case is far more dramatic in its clinical impact. A false‐negative case could escape a clarifying hysteroscopy because of the lack of support for the suspicion. As a consequence, the patient would not receive any tumour therapy. As can see in the ROC curve (Figure 7), a lower cut‐off may still give an acceptable specificity. The optimal cut‐off must be determined in a larger feasibility study so that false‐positive cases can be tolerated, but false‐negative cases no longer occur. As a result, the EC‐FISH Test could help to significantly reduce the proportion of unnecessary hysteroscopy, minimizing patient suffering.

If this EC‐FISH test was used as a preliminary test for suspected endometrial cancer, it could help reduce the very high proportion of hysteroscopies in which this suspicion is not confirmed. The negative predictive value of this EC‐FISH Test is 0.96. With a sensitivity of 0.91 and a specificity of 0.83 at the currently proposed preliminary cut‐off value, the test is not ready yet for clinical routine. The result of our study gives hope for a simple and cost‐effective FISH test for the exclusion of endometrial carcinoma, in which the specimen can be obtained just through a vaginal smear. Firstly, this would benefit the patients by not being scheduled for an invasive procedure. Secondly, the healthcare providers would have to spend less money on these procedures. But before this can become clinical standard, further studies on a larger cohort of EC from different centres are warranted.

## AUTHOR CONTRIBUTIONS

**Jörg Weimer:** Conceptualization (lead); data curation (lead); formal analysis (lead); methodology (lead); project administration (lead); supervision (lead); writing – original draft (lead); writing – review and editing (equal). **Martje Hüttmann:** Investigation (equal); validation (lead). **Asiyan Nusilati:** Investigation (lead). **Svenja Andreas:** Investigation (equal). **Jona Röseler:** Resources (supporting). **Nils Tribian:** Resources (supporting). **Christoph Rogmans:** Resources (supporting). **Matthias Bernhard Stope:** Resources (lead); writing – review and editing (lead). **Edgar Dahl:** Writing – review and editing (lead). **Alexander Mustea:** Resources (lead). **Elmar Stickeler:** Writing – review and editing (supporting). **Nina Hedemann:** Writing – original draft (supporting). **Inken Flörkemeier:** Writing – review and editing (supporting). **Katharina Tiemann:** Resources (lead). **Svetlana Magadeeva:** Investigation (supporting); writing – review and editing (supporting). **Astrid Dempfle:** Formal analysis (supporting); writing – review and editing (supporting). **Norbert Arnold:** Conceptualization (lead); supervision (supporting). **Nicolai Maass:** Funding acquisition (lead); resources (lead); supervision (supporting). **Dirk Bauerschlag:** Conceptualization (lead); methodology (supporting); project administration (supporting); supervision (lead); writing – review and editing (lead).

## CONFLICT OF INTEREST

The authors declare that there is no conflict of interest.

## Supporting information


Table S1

Appendix S1

## Data Availability

The data that support the findings of this study are available from the corresponding author upon reasonable request.
