# Supplementary material for: Fluorescence in situ hybridization test for detection of endometrial carcinoma cells by non‐invasive vaginal swab
Source: J Cell Mol Med. 2023 Jan 10;27(3):379–91. doi: 10.1111/jcmm.17658 (PMC9889703; doi:10.1111/jcmm.17658)
Supplement: Supplementary file 2 — Appendix S1 [file JCMM-27-379-s002.docx]

Table S1

Data of recounted fluorescence in situ hybridizations of the ten true positive cases listed in Table 2. The tables list the number of fluorescence signals per cell nucleus and the assessment derived from this.
